# MicroRNA-361-5p Inhibits Tumorigenesis and the EMT of HCC by Targeting Twist1

**DOI:** 10.1155/2020/8891876

**Published:** 2020-12-17

**Authors:** Liang-Chun Yin, Gang Xiao, Rui Zhou, Xiao-Ping Huang, Ning-Lei Li, Can-Liang Tan, Feng-Jiao Xie, Jun Weng, Li-Xin Liu

**Affiliations:** ^1^Department of General Surgery, The Third Affiliated Hospital of Southern Medical University, Guangzhou, Guangdong 510630, China; ^2^Second Department of Hepatobiliary Surgery, Zhujiang Hospital, State Key Laboratory of Organ Failure Research, Co-Innovation Center for Organ Failure Research, Southern Medical University, Guangzhou, Guangdong 510280, China

## Abstract

MicroRNA-361-5p (miR-361-5p) is a tumor suppressor miRNA that is dysregulated in several types of human cancer. However, the functional significance of miR-361-5p in hepatocellular carcinoma (HCC) is unclear. This study explored the biological function of miR-361-5p in regulating the progression of HCC and the underlying molecular mechanism. RT-qPCR analysis showed that miR-361-5p was downregulated in HCC tissues and cell lines. Functional analysis revealed that miR-361-5p acted as a tumor suppressor, inhibiting cell proliferation, migration, and invasion in HCC cell lines. Bioinformatics analyses identified Twist1 as a direct target of miR-361-5p, which was validated by dual-luciferase reporter assays, RT-qPCR, and western blotting. Rescue experiments indicated that Twist1 may mediate the tumor-suppressive effect of miR-361-5p in HCC cells, and this was supported by the effect of miR-361-5p on inhibiting the epithelial-mesenchymal transition (EMT) by targeting Twist1. This study is the first to suggest that miR-361-5p inhibits tumorigenesis and EMT in HCC by targeting Twist1. These findings are valuable for the diagnosis and clinical management of HCC.

## 1. Introduction

Hepatocellular carcinoma (HCC) is the fifth most common cancer worldwide and a major threat to human health [1–3]. Despite advances in the treatment of HCC in recent decades, HCC remains the third leading cause of cancer-related mortality worldwide, with nearly 600,000 deaths from HCC each year [4, 5]. Although liver resection and transplantation are the most common treatments for HCC, high rates of recurrence and metastasis lead to poor prognosis [6]. Therefore, early diagnosis and treatment of HCC are essential. Many genes related to the occurrence and metastasis of HCC have been identified; however, certain characteristics of HCC, such as heterogeneous differentiation and multifocal growth, hinder the development of new treatment methods for HCC.

MicroRNAs (miRNAs) are a class of small noncoding RNAs that regulate gene expression by promoting mRNA degradation or by inhibiting mRNA translation [7]. The microRNAs inhibit gene expression via by binding to the 3′-UTR (3′-untranslated region) of the target mRNA [8–11]. The miRNAs can regulate cancer cell proliferation, differentiation, apoptosis, invasion, migration, and angiogenesis by inhibiting the translation or transcription of their target mRNAs [12]. In recent years, miRNAs have received increasing attention for their potential use in cancer diagnosis and treatment [13]. The miR-361-5p is involved in tumor suppression in various cancers including colorectal cancer [14], gastric cancer [15], and prostate cancer [16]. However, the functional significance of miR-361-5p in HCC remains unclear. This study investigated the biological function of miR-361-5p in regulating the progression of HCC and explored the underlying molecular mechanism.

Twist1 is a well-known transcription factor that belongs to a highly conserved family of basic helix-loop-helix (bHLH) proteins [17]. It plays pivotal roles in embryonic development, and it is involved in tumor growth and metastasis [18]. Twist1 regulates tumor metastasis by inducing the epithelial-mesenchymal transition (EMT), a process in which epithelial cells acquire the motile and migratory characteristics of mesenchymal cells [19]. The EMT is a complex process that controls various biological programs in malignant tumors, especially drug resistance [20]. Multiple transcription factors (e.g., Zeb, Snail, Slug, and Twist) play key regulatory roles in the EMT process [20]. Twist1 is a negative regulator of epithelial gene expression (E-cadherin) and a positive regulator of mesenchymal gene expression (vimentin and N-cadherin), thereby inducing EMT. Abnormal expression of Twist1 leads to loss of E-cadherin-mediated intercellular adhesion, activation of mesenchymal markers, and induction of cell movement [21]. However, some aspects of the EMT remain unclear, and further research is needed to identify EMT-related biomarkers and develop targeted therapies. Abnormal expression of Twist1 is frequently observed in many types of cancer [22–25]. Twist1 is upregulated in HCC cell lines and promotes the proliferation and metastasis of HCC cells [22, 26]. In previous work from our group, we showed that Twist1 contributes to HCC cell migration and invasion [27]. Research on Twist1 has broad application prospects and potential therapeutic value in HCC. Bioinformatics analysis identified Twist1 as a possible direct target gene of miR-361-5p. However, the relationship between miR-361-5p and Twist1 in HCC is unclear.

In this study, we investigated the potential function of miR-361-5p in HCC for the first time. We also explored the mechanism underlying the effect of miR-361-5p on inhibiting the EMT by targeting Twist1 in HCC. This study elucidated a new molecular mechanism underlying HCC progression, thereby providing new insight for the diagnosis and treatment of HCC.

## 2. Materials and Methods

### 2.1. Tissue Specimens

Tissue specimens including tumor tissues and matched adjacent nontumor tissues were obtained from 30 HCC patients in the Department of General Surgery, The Third Affiliated Hospital of Southern Medical University (Guangzhou, China). None of the patients received chemotherapy or radiotherapy before surgery. The study was approved by the Ethics Committee of The Third Affiliated Hospital of Southern Medical University. All human tissues were obtained with informed consent. The samples were immediately frozen in liquid nitrogen and stored at -80°C. The tumor differentiation stage was defined according to the Edmondson-Steiner grading system. Of 30 HCC tissue samples, eight were poorly differentiated, 10 were medium-differentiated, and 12 were well-differentiated.

### 2.2. Western Blot Analysis

Cells were lysed with Radio Immunoprecipitation Assay lysis buffer (Beyotime, Shanghai, China). Proteins were separated by 10% sodium dodecyl sulphate-polyacrylamide gel electrophoresis and electro-transferred onto polyvinylidene difluoride membranes (Millipore, Billerica, MA, USA). After blocking with 5% nonfat dry milk, the membranes were incubated with primary antibodies (anti-Twist1, anti-E-cadherin, anti-vimentin, and anti-GAPDH; purchased from Santa Cruz Biotechnology, Santa Cruz, CA, USA) overnight at 4°C. Then, the membranes were washed and incubated with the corresponding secondary antibodies (Bosis, Beijing, China) for 1 h at 37°C. Protein bands were visualized using an ECL western blotting kit (Pierce Biotechnology, Waltham, MA, USA).

### 2.3. Immunohistochemistry

The expression of Twist1 in HCC tissues was detected by immunohistochemistry. Tissue specimens were fixed with 10% paraformaldehyde and embedded in paraffin wax. The samples were deparaffinized in xylene, rehydrated, and blocked with 5% goat serum. Then, the samples were incubated with anti-Twist1 antibody for 1–2 h at 37°C, washed three times with phosphate-buffered saline (PBS) for 5 min, and incubated with the corresponding secondary antibodies followed by a mixture of diaminobenzidine (DAB) for 1 h at 37°C. The slides were visualized using a microscope (Olympus, Tokyo, Japan).

### 2.4. In Situ Hybridization (ISH)

The ISH technique was performed to detect the expression of miR-361-5p in HCC. The ISH Detection Kit (Boster Bio-Engineering Company, Wuhan, China) and the ISH probe used for detecting miR-361-5p-labelled digoxin (Exiqon, Shanghai, China) were used. The samples were deparaffinized, hydrated, washed with PBS for 20 min, and incubated in prehybridization solution at 37°C for 3 h. Then, 5′-digoxigenin-labeled probe diluted in hybridization buffer was added to the sections at 37°C overnight. Tissues were then incubated with biotinylated mouse anti-digoxigenin antibody at 37°C for 60 min. After washing, the tissues were incubated with streptavidin-biotin-peroxidase complex at 37°C for 20 min. The slides were visualized with DAB followed by nuclear fast red counterstaining. Finally, the slides were analyzed under a microscope.

### 2.5. Cell Culture

The human HCC cell lines Hep3B, HepG2, and MHCC-97H and the normal human hepatic cell line LO2 were purchased from Shanghai Cell Bank of the Chinese Academy of Science (Shanghai, China). Cells were cultured in Dulbecco's Modified Eagle's Medium (DMEM, Gibco, Rockville, MD, USA) containing 10% fetal bovine serum (FBS, Gibco) at 37°C in an atmosphere with 5% CO_2_.

### 2.6. Transfection

The miR-361-5p mimics, miR-361-5p inhibitor, Twist1 siRNA, and their respective negative controls were obtained from RiboBio (Guangzhou, China) and separately transfected into HCC cell lines using Lipofectamine 2000 (Invitrogen, Carlsbad, CA, USA) according to the manufacturer's protocols. After 48 h of transfection, the cells were harvested and used in experiments.

### 2.7. Reverse Transcription-Quantitative Polymerase Chain Reaction (RT-qPCR)

Total RNA was extracted using the TRIzol reagent (Promega, Madison, WI, USA) according to the manufacturer's protocols. For miR-361-5p detection, 100 ng total RNA was reverse transcribed into cDNA with specific primers for miR-361-5p analysis using the First Stand cDNA Synthesis Kit (Tiangen Biotechnology, Beijing, China). For Twist1 detection, cDNA was synthesized by M-MLV reverse transcriptase (Takara, Dalian, China). Quantitative RT-PCR was performed using a SYBR Green PCR Master Mix kit (Applied Biosystems, Foster City, CA, USA) on an Applied Biosystems 7300 Real-Time PCR System (Thermo Fisher Scientific, Inc., Waltham, MA, USA). Relative expression was calculated using the 2^-*ΔΔ*Ct^ method. GAPDH and U6 were used as controls for Twist1 and miR-361-5p. The RT-qPCR primers were as follows: miR-361-5p forward, 5′-ATAAAGTGCTGACAGTGCAGATAGTG-3′, and reverse, 5′-TCAAGTACCCACAGTGCGGT-3′; U6 forward, 5′-CTCGCTTCGGCAGCACA-3′, and reverse, 5′-AACGCTTCACGAATTTGCGT-3′; Twist1 forward, 5′-CCAGGTACATCGACTTCCTCTA-3′, and reverse, 5′-CCATCCT CCAGACCGAGAA-3′; GAPDH forward, 5′-CCATGTTCGTCATGGGTGTG-3′, and reverse, 5′-GGTGCTAAGCAGTTGGTGGTG-3′.

### 2.8. Cell Counting Kit-8 (CCK-8) Assay

The CCK-8 assay was performed to measure cell proliferation according to the manufacturer's instructions. In brief, cultured cells were plated and incubated in 96-well plates at 3 × 10^4^ cells/well for 0, 24, 48, and 72 h. Then, 10 *μ*L CCK-8 solution (Dojindo Molecular Technologies, Kumamoto, Japan) was added to each well. Proliferation rates were measured with a microplate reader (Molecular Devices, Eugene, OR, USA) at an absorbance of 450 nm at 24, 48, and 72 h.

### 2.9. Transwell Assay

Cell migration and invasion were measured using the Transwell assay. Transwell chambers (8 *μ*m pore size; Millipore) with and without Matrigel (BD Biosciences, Billerica, MA, USA) were used in the Transwell assay. Cells were seeded in the upper chambers with serum-free medium, and 10% FBS was added to the lower chambers. The upper chambers were coated with Matrigel for the cell invasion assay, whereas the cell migration assay was performed without Matrigel. The migrated or invaded cells were fixed with methanol, stained with 0.1% Crystal Violet (Sigma-Aldrich) after 24 h at 37°C, and counted under a microscope (Olympus, Tokyo, Japan).

### 2.10. Dual-Luciferase Reporter Assay

A dual-luciferase reporter assay was performed to validate Twist1 as a direct target of miR-361-5p. The 3′-UTR sequence of wild-type (WT) or mutant-type (Mut) Twist1 was inserted into the pmirGLO Dual-Luciferase vector (Promega, Madison, WI, USA). Then, the pmirGLO vector carrying miR-361-5p mimics and the Twist1 3′-UTR was transfected into HCC cells using Lipofectamine 2000 (Invitrogen) and incubated for 48 h. A Dual-Luciferase reporter assay system kit (Promega) was used to analyze the relative luciferase activity.

### 2.11. Rescue Assay

Twist1-overexpressing plasmid and miR-361-5p mimics were cotransfected into HCC cell lines using Lipofectamine 2000. After 48 h, the Twist1 expression was detected by RT-qPCR.

### 2.12. Statistical Analysis

All statistical analyses were performed with the SPSS 22.0 software (SPSS Inc., Chicago, IL, USA) and GraphPad Prism 5 (GraphPad Software, Inc., San Diego, CA, USA). Data were presented as the mean ± standard deviation from at least three independent experiments. Differences were analyzed by Student's *t*-test or one-way ANOVA with Bonferroni post hoc test. Statistical significance was determined at ^∗^*P* < 0.05, ^∗∗^*P* < 0.01, and ^∗∗∗^*P* < 0.001.

## 3. Results

### 3.1. The miR-361-5p Is Downregulated in HCC and Correlated with Poor Survival

**Figure 1:**
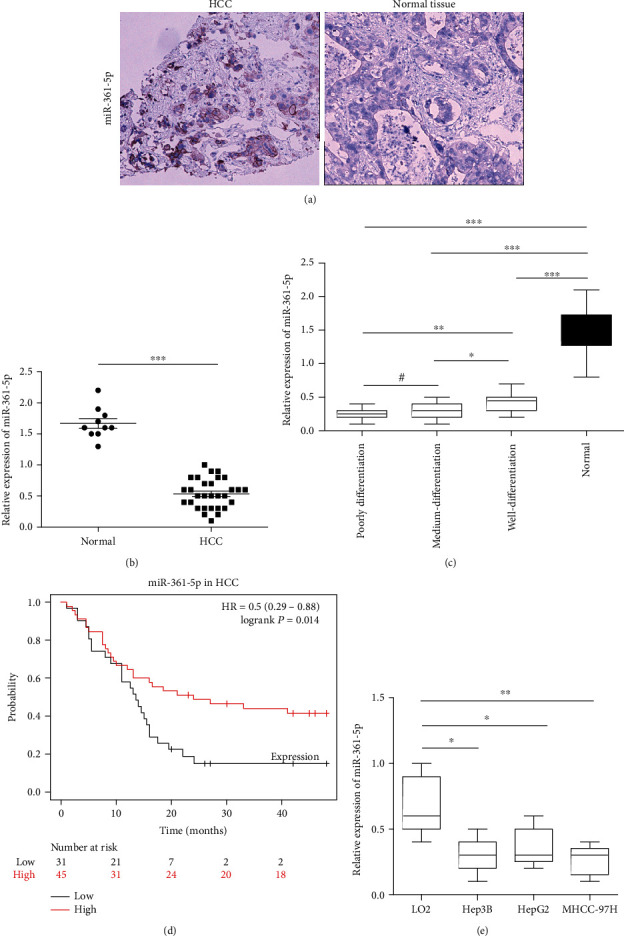
miR-361-5p is downregulated in HCC and correlated with poor survival. (a) Representative staining images of miR-361-5p in HCC tumor tissues and adjacent nontumorous tissues with ISH (×200 magnification). (b) RT-qPCR analysis of miR-361-5p in HCC tissues and adjacent nontumorous tissues. (c) RT-qPCR analysis of miR-361-5p in poorly differentiated HCC, medium-differentiated HCC, well-differentiated HCC, and normal live tissues. (d) Association between miR-361-5p and survival of HCC patients was explored by Kaplan-Meier analysis. (e) RT-qPCR analysis of miR-361-5p in HCC cell lines (Hep3B, HepG2, and MHCC-97H) and in normal human hepatic LO2 cells. The results were presented as means ± SD. Statistical significance was determined at ^∗^*P* < 0.05, ^∗∗^*P* < 0.01, and ^∗∗∗^*P* < 0.001; # represents no statistical significance.

To determine the biological role of miR-361-5p in HCC, the miR-361-5p expression in HCC tissues and cell lines was detected by RT-qPCR, and its expression in HCC tumor tissues and adjacent nontumorous tissues was detected by ISH (Figure 1(a)). The results showed that the miR-361-5p expression was significantly lower in HCC tissues than in nontumor tissues (Figure 1(b)) and significantly lower in poorly differentiated than in well-differentiated HCC tissues (Figure 1(c)). Kaplan-Meier analysis showed that the clinical outcomes of patients with the high miR-361-5p expression were better than those of patients with the low miR-361-5p expression (*P* = 0.014) (Figure 1(d)). The miR-361-5p expression was markedly lower in HCC cell lines (Hep3B, HepG2, and MHCC-97H) than in normal human hepatic LO2 cells (Figure 1(e)). These results indicated that miR-361-5p was downregulated in HCC and correlated with poor survival.

### 3.2. The miR-361-5p Inhibits Cell Proliferation, Migration, and Invasion in HCC Cells

**Figure 2:**
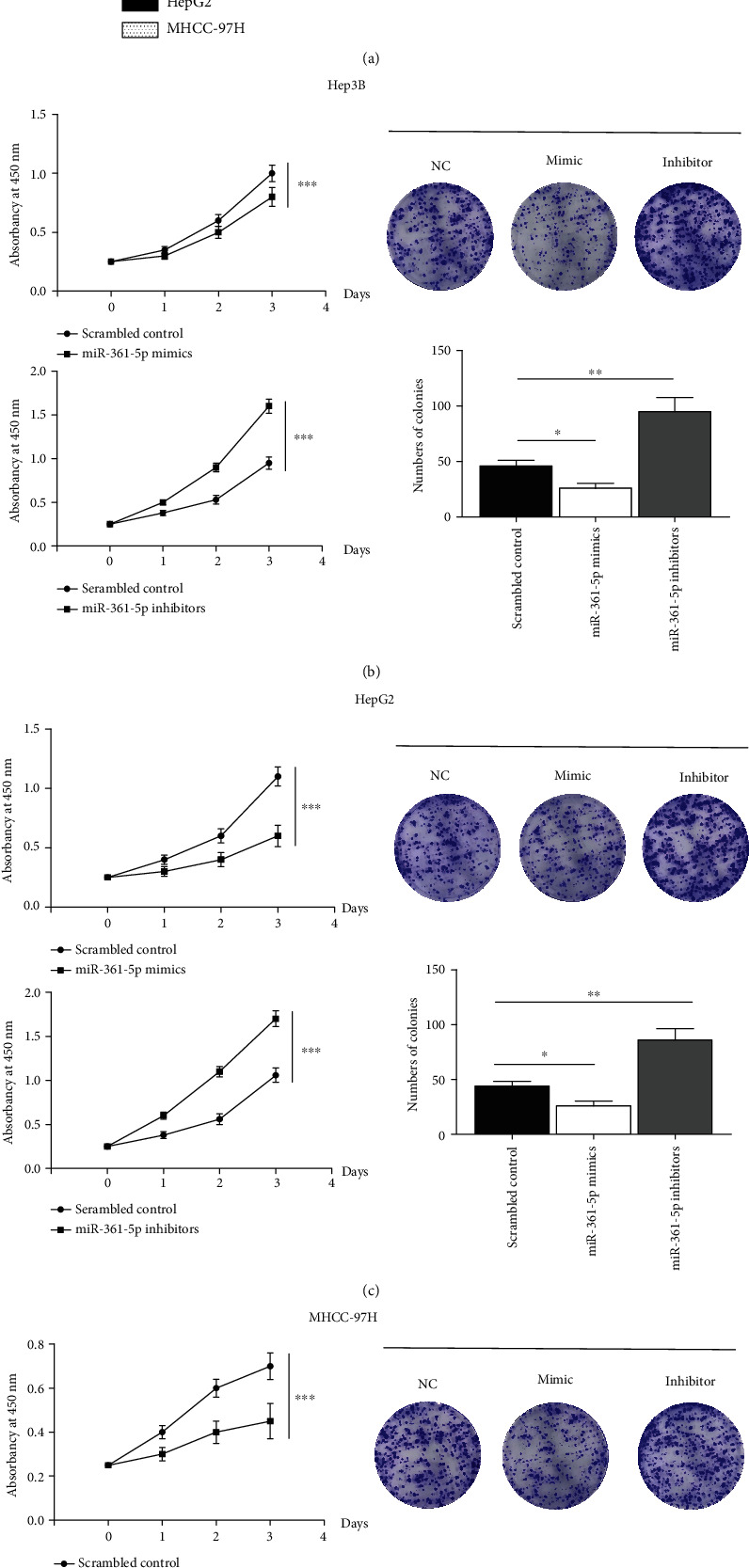
miR-361-5p inhibits HCC cell proliferation. (a) RT-qPCR analysis of miR-361-5p in Hep3B, HepG2, and MHCC-97H cells transfected with miR-361-5p mimics or miR-361-5p inhibitors. The CCK-8 assay was used to measure the proliferation ability of the Hep3B (b), HepG2 (c), and MHCC-97H (d) cells transfected with miR-361-5p mimics or miR-361-5p inhibitors. The results were presented as means ± SD. Statistical significance was determined at ^∗^*P* < 0.05, ^∗∗^*P* < 0.01, and ^∗∗∗^*P* < 0.001.

**Figure 3:**
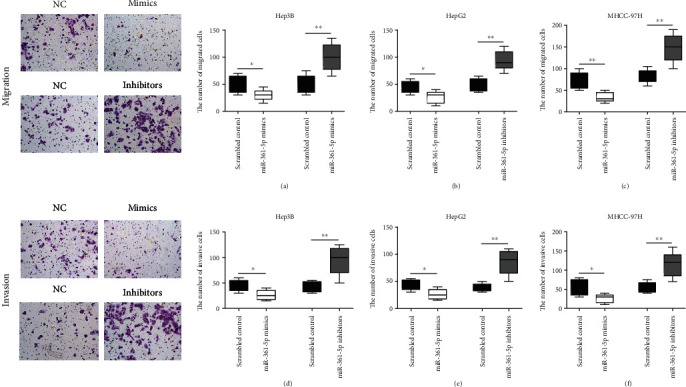
miR-361-5p inhibits HCC cell migration and invasion. Transwell assay was used to measure the migration and invasion ability of the Hep3B (a, d), HepG2 (b, e), and MHCC-97H (c, f) cells transfected with miR-361-5p mimics or miR-361-5p inhibitors. The results were presented as means ± SD. Statistical significance was determined at ^∗^*P* < 0.05 and ^∗∗^*P* < 0.01.

To investigate the role of miR-361-5p in HCC progression, Hep3B, HepG2, and MHCC-97H cells were transfected with miR-361-5p mimics or miR-361-5p inhibitors. The miR-361-5p mimics increased the miR-361-5p expression, whereas miR-361-5p inhibitors decreased the miR-361-5p expression (Figure 2(a)). Overexpression of miR-361-5p significantly repressed cell proliferation, whereas downregulation of miR-361-5p significantly promoted cell proliferation in Hep3B (Figure 2(b)), HepG2 (Figure 2(c)), and MHCC-97H (Figure 2(d)) cells. Assessment of the role of miR-361-5p in HCC cell migration and invasion showed that overexpression of miR-361-5p significantly repressed cell migration and invasion, whereas downregulation of miR-361-5p significantly promoted cell migration and invasion in Hep3B (Figures 3(a) and 3(d)), HepG2 (Figures 3(b) and 3(e)), and MHCC-97H (Figures 3(c) and 3(f)) cells. Collectively, these data suggested that miR-361-5p acted as a tumor suppressor in HCC.

### 3.3. The miR-361-5p Inhibits Twist1 Expression by Directly Binding to Its 3′-UTR in HCC

**Figure 4:**
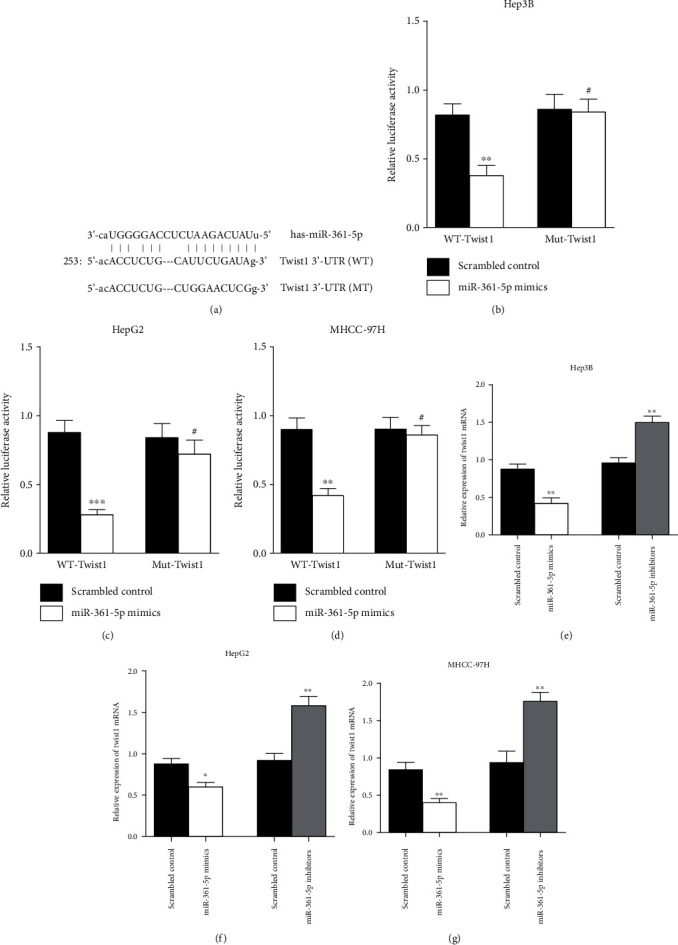
miR-361-5p inhibits the Twist1 expression by directly binding to its 3′-UTR in HCC. (a) The bioinformatics analyses of the binding sites between miR-361-5p and the 3′-UTR of Twist1. The luciferase activity of Hep3B (b), HepG2 (c), and MHCC-97H (d) cells was detected after cotransfection with a control vector or miR-361-5p mimics and a luciferase reporter construct containing the wild-type (WT) or mutant-type (MT) 3′-UTR of Twist1. The mRNA expression levels of Twist1 in Hep3B (e), HepG2 (f), and MHCC-97H (g) cells containing miR-361-5p mimics or miR-361-5p inhibitors were detected by RT-qPCR analysis. The results were presented as means ± SD. Statistical significance was determined at ^∗^*P* < 0.05, ^∗∗^*P* < 0.01, and ^∗∗∗^*P* < 0.001; # represents no statistical significance.

To further understand the molecular basis of miR-361-5p, it is important to identify an mRNA target of miR-361-5p that may be implicated in the regulation of miR-361-5p in HCC. Bioinformatics analyses were performed to predict the possible targets of miR-361-5p. The target prediction programs TargetScan (http://www.targetscan.org/) and miRBase (http://www.mirbase.org/) indicated that the 3′-UTR of Twist1 contains a putative binding sequence for miR-361-5p (Figure 4(a)). The bioinformatics analyses indicated that Twist1 is a potential direct target gene of miR-361-5p. Previous work from our group shows that Twist1 is closely involved in HCC cell migration and invasion [27]. Therefore, we performed a dual-luciferase reporter assay to confirm that Twist1 is a target of miR-361-5p. The results showed that miR-361-5p overexpression inhibited luciferase activity in cells transfected with the luciferase reporter vector containing the wild-type (WT) Twist1 3′-UTR but not in those transfected with the mutant-type (MT) Twist1 3′-UTR in Hep3B (Figure 4(b)), HepG2 (Figure 4(c)), and MHCC-97H (Figure 4(d)) cells. These results suggested that miR-361-5p inhibited the Twist1 expression by directly binding to the 3′-UTR of Twist1. To confirm these results, the Twist1 expression was assessed in HCC cell lines transfected with miR-361-5p mimics or inhibitors by RT-qPCR. The results showed that miR-361-5p mimics downregulated, whereas miR-361-5p inhibitors upregulated the mRNA expression of Twist1 in Hep3B (Figure 4(e)), HepG2 (Figure 4(f)), and MHCC-97H (Figure 4(g)) cells. Taken together, the results indicated that miR-361-5p directly targeted the 3′-UTR of Twist1 and inhibited the Twist1 expression in HCC.

### 3.4. Twist1 Is Upregulated in HCC and Promotes Cell Proliferation, Migration, and Invasion in HCC Cells

**Figure 5:**
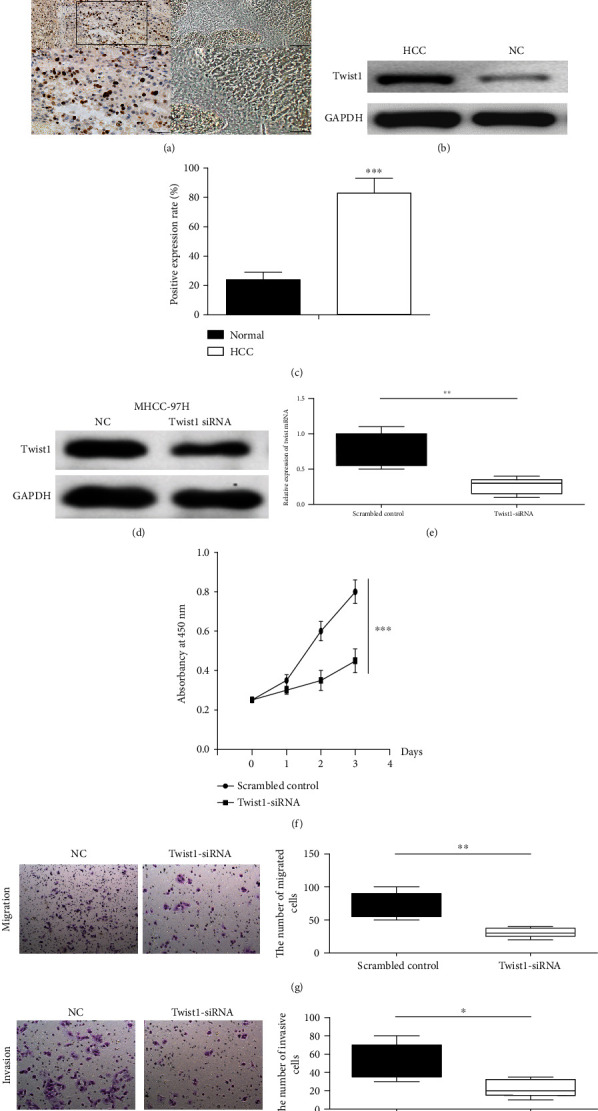
Twist1 is upregulated in HCC and promotes cell proliferation, migration, and invasion in HCC cells. (a) Representative images of Twist1 protein in HCC tissues and adjacent nontumorous tissues with immunohistochemistry. (b) Western blot analysis of Twist1 in HCC. (c) The positive expression rate of Twist1 in HCC. Knockdown of Twist1 suppressed HCC cell growth. The protein (d) and relative mRNA (e) expression levels of Twist1 in MHCC-97H cells transfected with siRNA against Twist1 were investigated by western blot analysis and RT-qPCR. The cell proliferation (f), migration (g), and invasion (h) abilities were measured in MHCC-97H cells. The results were presented as means ± SD. Statistical significance was determined at ^∗^*P* < 0.05, ^∗∗^*P* < 0.01, and ^∗∗∗^*P* < 0.001.

To determine the biological role of Twist1 in HCC, the expression of Twist1 in HCC tissues was detected by IHC (Figure 5(a)) and western blotting (Figure 5(b)). The results showed that the positive expression rate of Twist1 was significantly higher in HCC tissues than in nontumor tissues (Figure 5(c)). To investigate the function of Twist1 in HCC progression, MHCC-97H cells were transfected with small interfering RNA against Twist1, which downregulated Twist1 at the protein (Figure 5(d)) and mRNA (Figure 5(e)) levels. Downregulation of Twist1 inhibited cell proliferation (Figure 5(f)), migration (Figure 5(g)), and invasion (Figure 5(h)) in MHCC-97H cells. Collectively, these data suggested that Twist1 acted as a tumor promoter in HCC.

### 3.5. Twist1 Mediates the Tumor-Suppressive Effect of miR-361-5p in HCC Cells

**Figure 6:**
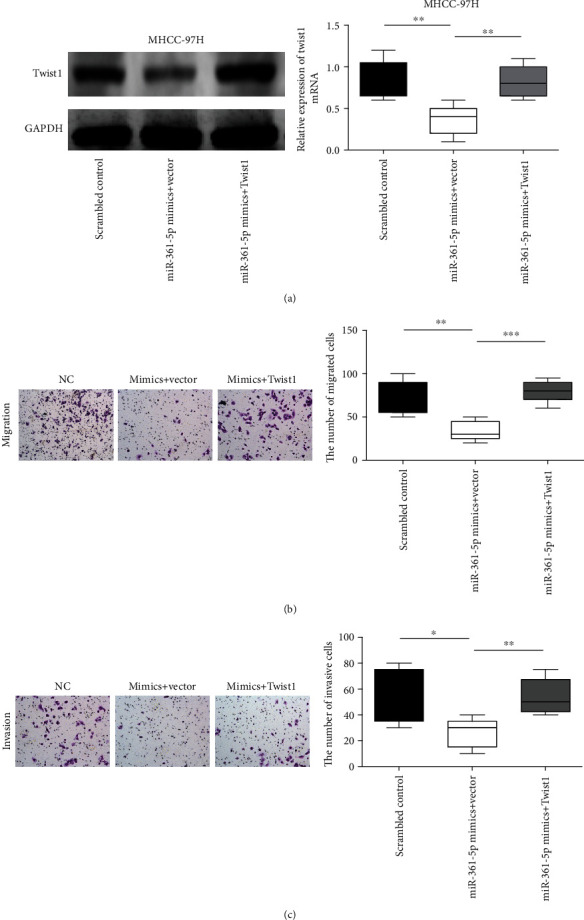
Overexpression of Twist1 rescues the tumor-suppressive effect of miR-361-5p in HCC cells. MHCC-97H cells were cotransfected with Twist1-overexpressing plasmid and miR-361-5p mimics. The mRNA and protein expression of Twist1 in MHCC-97H cells were detected by RT-qPCR and western blotting (a). Transwell assay was used to measure the migration (b) and invasion (c) ability of the MHCC-97H cells. The results were presented as means ± SD. Statistical significance was determined at ^∗^*P* < 0.05, ^∗∗^*P* < 0.01, and ^∗∗∗^*P* < 0.001.

To confirm the functional relevance of miR-361-5p targeting Twist1, a rescue assay was performed. MHCC-97H cells were cotransfected with Twist1-overexpressing plasmid and miR-361-5p mimics. The results showed that Twist1 overexpression largely restored the mRNA and protein expression of Twist1 reduced by miR-361-5p mimics in MHCC-97H cells (Figure 6(a)). Twist1 overexpression also restored the inhibitory effects of miR-361-5p on cell migration (Figure 6(b)) and invasion (Figure 6(c)) in MHCC-97H cells. These results suggested that Twist1 overexpression rescued the inhibitory effects of miR-361-5p in HCC.

### 3.6. The miR-361-5p Inhibits EMT of HCC Cells by Targeting Twist1

**Figure 7:**
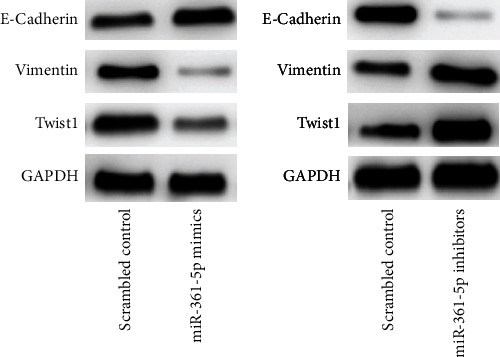
miR-361-5p inhibits EMT of HCC cells by targeting Twist1. The protein expressions of E-cadherin, vimentin, and Twist1 in MHCC-97H cells with miR-361-5p mimics or miR-361-5p inhibitors were investigated by western blot analysis.

Finally, to explore the mechanism underlying the inhibitory effect of miR-361-5p on HCC, we investigated the role of miR-361-5p and Twist1 in EMT. The miR-361-5p overexpression upregulated E-cadherin and downregulated vimentin and Twist1 expression in MHCC-97H cells (Figure 7), whereas miR-361-5p inhibition had the opposite effect (Figure 7). These results suggested that miR-361-5p inhibited EMT in HCC by targeting Twist1.

## 4. Discussion

The miRNAs have important molecular functions and pathological significance in HCC [28–30]. Therefore, identifying miRNAs that may serve as markers for the diagnosis, prognosis, and treatment of HCC is critical. In this study, we showed that miR-361-5p was downregulated in HCC tissues and cell lines, and miR-361-5p acted as a tumor suppressor, inhibiting cell proliferation, migration, and invasion in HCC cell lines. In addition, this study is the first to demonstrate that miR-361-5p directly targeted the 3′-UTR of Twist1 and inhibited the EMT in HCC cells by targeting Twist1. These results suggested that miR-361-5p played an important role in HCC and may be a novel therapeutic target in HCC.

The miR-361-5p is a tumor-suppressor miRNA that is dysregulated in several types of human cancer. Downregulation of miR-361-5p is associated with prostate cancer [31, 32], breast cancer [33], papillary thyroid carcinoma [34], lung cancer [35, 36], and colorectal and gastric cancers [15, 37]. The present results showed that miR-361-5p was downregulated in HCC and correlated with poor survival, which was consistent with previous reports (Figures 1(b)–1(d)). Overexpression of miR-361-5p inhibits proliferation, migration, and invasion in colorectal and gastric cancer cells [15]. The miR-361-5p inhibits the growth and proliferation of HCC cells by targeting CXCR6 [38]. In addition, miR-361-5p was shown to inhibit proliferation and invasion of HCC cells by targeting VEGFA [39]. Consistently, we showed that overexpression of miR-361-5p significantly repressed cell proliferation, migration, and invasion, whereas downregulation of miR-361-5p significantly promoted cell proliferation, migration, and invasion in Hep3B, HepG2, and MHCC-97H cells (Figures 2 and 3). In addition, miR-361-5p inhibits the EMT in glioma cells [40], which is consistent with the present results (Figure 7).

In this study, we performed bioinformatics analysis to predict possible targets of miR-361-5p. The target prediction databases suggested that Twist1 was a direct target of miR-361-5p (Figure 4(a)). This prediction was validated by dual-luciferase reporter assay and RT-qPCR (Figure 4). Rescue experiments showed that Twist1 overexpression restored the inhibitory effects of miR-361-5p on cell migration (Figure 6(b)) and invasion (Figure 6(c)) in MHCC-97H cells. These results suggested that Twist1 mediated the tumor-suppressive effect of miR-361-5p in HCC cells. Twist1 is upregulated in many types of cancer such as HCC [22], gastric cancer [23], breast cancer [24], prostate cancer [16], and lung cancer [25]. It plays a key role in regulating cancer progression and metastasis [18, 41]. Twist1 is upregulated in many HCC cell lines and promotes the proliferation and migration of HCC cells [22]. Consistently, we showed that deletion of Twist1 inhibited cell proliferation, migration, and invasion in HCC cells (Figures 5(f)–5(h)). A major function of Twist1 is to regulate tumor metastasis by modulating EMT [42, 43]. Twist1 promotes EMT by affecting downstream genes such as E-cadherin and vimentin [43]. Consistently, we showed that miR-361-5p overexpression upregulated E-cadherin and downregulated vimentin and Twist1 expression in MHCC-97H cells, whereas miR-361-5p inhibition had the opposite effect (Figure 7). Here, we showed that miR-361-5p inhibited EMT of HCC by targeting Twist1.

In addition to regulating the EMT process, Twist1 also regulates the expression of miRNAs as a downstream target of these miRNAs [44]. Multiple miRNAs act as negative regulators of Twist1 [45]. The Twist1/miR-584/TUSC2 pathway plays a role in the resistance to apoptosis in thyroid cancer cells [46]. The miR-16-1-3p suppresses A549 cell proliferation, migration, and invasion by targeting Twist1 [47]. The miR-186 inhibits cell proliferation, migration, invasion, and EMT by targeting Twist1 in cholangiocarcinoma [48]. The miR-539 overexpression suppressed the invasion, migration, and EMT of BXPC-3 and PANC-1 cells through targeting TWIST1 [49]. However, there are no studies identifying Twist1 as a direct target of miR-361-5p in HCC. In this study, we showed for the first time that miR-361-5p directly targeted Twist1 to regulate proliferation, migration, invasion, and the EMT in HCC cells.

In conclusion, this study is the first to show that miR-361-5p is downregulated in HCC tissues and cell lines. We also demonstrated that miR-361-5p inhibited tumorigenesis and the EMT of HCC by targeting Twist1. These findings have diagnostic value and potential clinical value in HCC.

## Data Availability

The data used to support the findings of this study are included within the article and the supplementary material files.
